# Across-trial spatial suppression in visual search

**DOI:** 10.3758/s13414-021-02341-x

**Published:** 2021-06-25

**Authors:** Lishuang Wang, Benchi Wang, Jan Theeuwes

**Affiliations:** 1 Key Laboratory of Brain, Cognition and Education Sciences, South China Normal University, Ministry of Education, Guangzhou, China; 2 Institute for Brain Research and Rehabilitation, South China Normal University, Zhongshan Road West 55, Guangzhou, 510000 China; 3 Center for Studies of Psychological Application, South China Normal University, Guangzhou, China; 4 Guangdong Key Laboratory of Mental Health and Cognitive Science, South China Normal University, Guangzhou, China; 5 Department of Experimental and Applied Psychology, Vrije Universiteit Amsterdam, Amsterdam, The Netherlands; 6 Institute Brain and Behavior Amsterdam (iBBA), Vrije Universiteit Amsterdam, Amsterdam, The Netherlands

**Keywords:** Attentional selection, Proactive suppression, Implicit, Priority map

## Abstract

In order to focus on objects of interest, humans must be able to avoid distraction by salient stimuli that are not relevant to the task at hand. Many recent studies have shown that through statistical learning we are able to suppress the location that is most likely to contain a salient distractor. Here we demonstrate a remarkable flexibility in attentional suppression. Participants had to search for a shape singleton while a color distractor singleton was present. Unbeknown to the participant, the color distractor was presented according to a consistent pattern across trials. Our findings show that participants learn this distractor sequence as they proactively suppressed the anticipated location of the distractor on the next trial. Critically, none of the participants were aware of these hidden sequences. We conclude that the spatial priority map is highly flexible, operating at a subconscious level preparing the attentional system for what will happen next.

## Introduction

In everyday life it is important that we are able to resist all distraction by objects and events that grab our attention. For example, we may try to ignore the flashing banner on the side of the computer screen while trying to read that interesting article, or ignore the dynamically moving billboard along the highway while trying to keep your eyes on the road. It is known that objects that are salient and stand out from the environment have the ability to capture our attention even when we are trying to do something else (Theeuwes, 1991, 1992). Recently, there has been a surge in research investigating how and under what conditions we are able to avoid distraction by salient objects and events (Chang & Egeth, 2019, 2020; Failing et al., 2019; Feldmann-Wüstefeld et al., 2015; Feldmann-Wüstefeld et al., 2019; Ferrante et al., 2018; Gaspelin et al., 2015, 2017; Gaspelin & Luck, 2018a, 2018b, 2019; Stilwell et al., 2019; van Moorselaar & Slagter, 2019; Vatterott & Vecera, 2012; Wang & Theeuwes, 2018a, 2018b, 2018c; Won et al., 2019).

One way to accomplish the suppression of a salient object is by presenting it more often in one particular location than in all other locations (Ferrante et al., 2018; Wang & Theeuwes, 2018a, 2018b). In this case, participants learn to extract the statistical regularities of the display characteristics, which in turn biases attentional selection such that the location that is likely to contain a distractor becomes suppressed. This type of learning has been described as “statistical learning,” and refers to an implicit learning process that allows the extraction of distributional properties from sensory input across time and space (Aslin et al., 1998; Frost et al., 2015).

Even though previous studies have demonstrated that participants can easily extract the regularities regarding the (task-relevant) target (e.g., Chun & Jiang, 1998; Geng & Behrmann, 2005; Miller, 1988), recent studies have shown that observers can also learn the statistical regularities regarding task-irrelevant distractors (e.g., Wang & Theeuwes, 2018a). These findings indicate that people can learn not only from attending to relevant objects but also from ignoring objects that are irrelevant. For example, Wang and Theeuwes (2018a, 2018b) employed the classic additional singleton in which participants searched for a salient shape singleton (i.e., a diamond between circles or a circle between diamonds) while ignoring a colored distractor singleton. Unbeknown to the participants, the colored distractor singleton was systematically presented more often in one location than in all other locations. The results showed that the highly salient distractor singleton interfered less with search when it was presented at the high-probability location relative to all other locations.

Several studies indicated that the interference by the salient distractor was reduced because the location that was likely to contain a distractor was proactively suppressed (i.e., before display onset). For example, an electroencephalography (EEG) study by Wang and colleagues (Wang, van Driel, et al., 2019a) showed pre-stimulus (i.e., before display onset) enhanced parieto-occipital alpha power contralateral to the high-probability location, suggesting anticipated suppression. In addition, an eye-tracking study (Wang, Samara, & Theeuwes, 2019b) showed fewer saccades landing at the distractor when it was presented at the high-probability location than at a low-probability location. Huang and colleagues (Huang et al., 2021) showed slower reaction times to probes, which were presented before display onset, at the anticipated high-probability distractor location relative to the low-probability location. Learned suppression has also been observed in the non-spatial domain (see Geng et al., 2019). For example, a color singleton that initially captured attention no longer does so when it is repeatedly shown (Vatterott & Vecera, 2012). Moreover, distractor colors that appear with a high probability are suppressed more efficiently than low-probability distractor colors (Stilwell et al., 2019). In addition, feature-based and spatial-based statistical regularities interact as suppression of a distractor is more effective when presented in a location where its feature (e.g., its color) is more likely to be observed (Failing et al., 2019).

Because of proactive suppression, within the spatial priority map, locations that have a high probability of containing a distractor compete less for attention than all other locations (see also Ferrante et al., 2018; Goschy et al., 2014; Huang et al., 2021; Kong et al., 2020). Even though long-lasting biases within the spatial priority map have been well documented (Chelazzi et al., 2014; Zelinsky & Bisley, 2015), it is unknown whether the suppression can be altered on a trial-by-trial basis. The current study tested this notion by presenting on each trial the salient distractor at a new location, which followed a particular consistent pattern across trials (i.e., it moved clockwise or counter-clockwise) or was completely randomly placed. The question was whether participants would learn this consistent pattern and would suppress the anticipated location of the salient distractor. It would imply that the weights within the spatial priority map can be dynamically adapted from trial to trial such that the weight of the anticipated distractor location is reduced, resulting in reduced capture when the distractor is presented at the anticipated location.

Previous studies have shown that participants can learn to extract inter-trial statistical associations regarding subsequent target locations. For example, in Li and Theeuwes (2020) the target was always randomly placed on any of the eight locations on an imaginary circle around the fixation point. There were two exceptions: if the target was presented on the left side of the display it would be presented on the next trial on the right side, and if the target was presented on the top it would be presented at the bottom position on the next trial. The results showed that participants learned this regularity as they were significantly faster to respond to the target when presented at this anticipated location relative to a random location. Note, however, that this study was about anticipating the target location, which may be learned much easier as it is the object participants are looking for.

The current study investigated whether participants can learn to anticipate the location of the salient distractor when the location of the distractor moves across the display. If so, it would imply that the attentional system anticipates what will happen next and that suppression can be flexibly applied proactively to the anticipated location. It would provide evidence that the spatial priority map can be tuned on each and every trial to the anticipated learned occurrence of an event (i.e., in this case the location of anticipated salient distractor).

## Experiment 1

### Method

The study was approved by the Ethics Review Committee of South China Normal University (2020-3-013).

#### Participants

Forty-eight college students (six men and 42 women: with a mean age of 19.4 ± 1.5 years old) were recruited from South China Normal University in China for monetary compensation. All participants provided written informed consent before the study, and reported normal color vision and normal or corrected-to-normal visual acuity. They were evenly divided into two groups: one group was exposed to sequential learning; the other group did not receive any consistent sequences (baseline). Sample size was predetermined based on the critical interaction effect in the pilot study (16 per group), partial η^2^ = 0.26. With 24 subjects per group and alpha = 0.05, power for this effect should be larger than 0.9.

#### Apparatus and stimuli

**Fig. 1:**
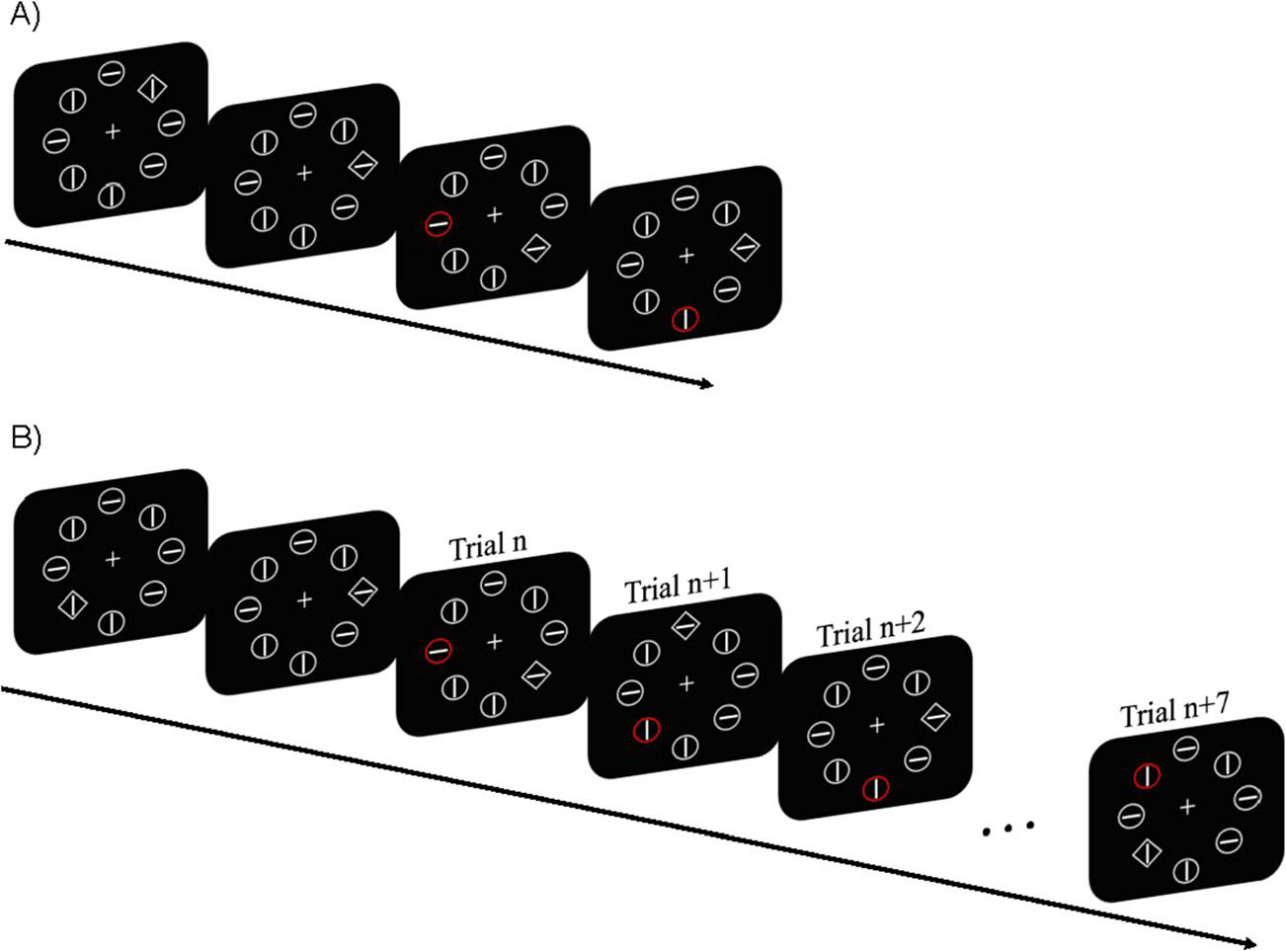
**a** Example of trial sequence in the baseline group: across trials, the location of the distractor is randomly picked. **b** Example of trial sequence in the learning group: across trials, the location of the distractor moves to the next position either in a clockwise or an anticlockwise way. For each test block, both groups also took Part in trials in which there was no distractor. These distractor-present and -absent trials were presented in separate mini-blocks to ensure that learning of the distractor location was not disrupted by trials in which there was no distractor present. Half of the group started with the distractor-absent trials, the other started with the distractor-present trials

Participants were seated in a dimly lit laboratory, 57 cm away from the liquid crystal display (LCD) color monitor with their chin on a chinrest. The background was black (Red-Green-Blue [RGB]: 0, 0, 0). As illustrated in Fig. 1a, the primary search display contained one unfilled diamond (subtended by 2° × 2°) among seven circles with a radius of 1°, or vice versa. Searching for a diamond or circle was counterbalanced across participants. In the distractor-absent condition, display elements were colored in gray (RGB: 128, 128, 128) and were centered 4.0° from the fixation (a white cross, 0.67° × 0.67°, RGB: 255, 255, 255), containing a vertical or horizontal gray line (0.3° × 1.5°). In the distractor-present condition, one of the display elements was colored in red (RGB: 255, 0, 0). Stimulus presentation and response registration were controlled by custom scripts written in Python 2.7 with the package Pygame (www.pygame.org).

#### Procedure and design

On each trial, the fixation cross remained present until the end of the trial. After 500 ms a search display was presented in which participants were required to search for a specific shape (for half of the participants a circle shape; for the other half a diamond shape), and to indicate whether the line segment inside the target was vertical or horizontal by pressing the up or left key on the keyboard as fast as possible. Warning messages were presented if the participants did not respond, or if the wrong key was pressed. The inter-trial interval (ITI) was 500 ms and 750 ms at random. The search target was present on each trial, and appeared equally often at each location. In distractor-present trials, one of the gray elements was colored red.

Baseline versus learning was manipulated between subjects: In the baseline group, the distractor location was randomly selected across trials (see Fig. 1a), a manipulation similar to the original Theeuwes (1992) task. In the learning group, the distractor location was presented according to a pre-set sequence in which the distractor moved to the adjacent location for the next trial consistently in either a clockwise or an anticlockwise way, the order of which was counterbalanced between subjects and kept the same within subjects (see Fig. 1b). Participants completed ten practice trials, followed by ten mini-blocks of 120 test trials. A mini-block of trials consisted of 80 trials in which a distractor was present and 40 trials in which the distractor was absent. These distractor-present and distractor-absent trials were presented in separate mini-blocks to ensure that learning of the distractor location was not disrupted in distractor present trials. Half of the participants started with the mini-block of distractor-absent trials, while the other half started with the mini-block of distractor-present trials. After the experiment, participants of the learning group were told that some elements in the display were consistently placed at particular locations within the display. Participants were asked whether they noticed these regularities, and if they did, whether they could tell what they had noticed.

### Results

Trials on which the response times (RTs) were faster than 200 ms or slower than 1,600 ms and trials on which no response was given (1.5% and 2.1% for baseline and learning groups, respectively) were excluded from analyses. Note that, the pattern of results remained the same when slightly different outlier standards (e.g., slower than 1,700 ms) were applied.

**Fig. 2:**
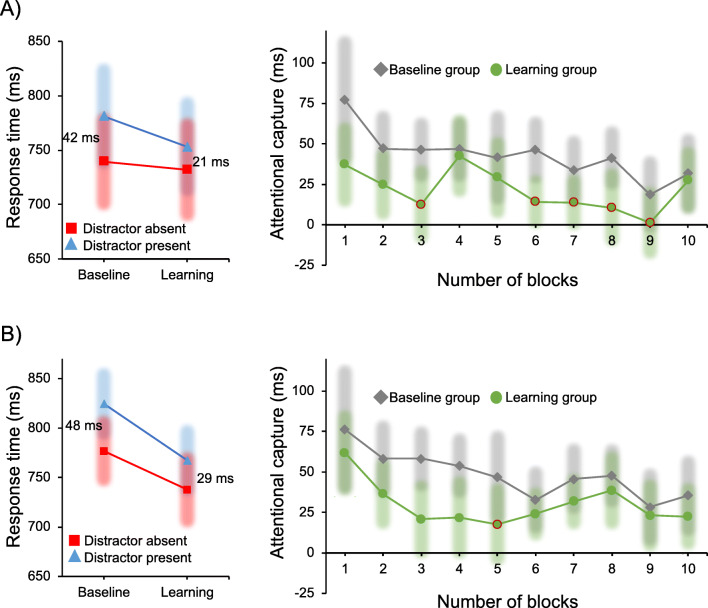
Results in Experiments 1 (**a**) and 2 (**b**). Red outlines in the right panels indicate the block in which the attentional capture effect was completely eliminated (*p* > .05). Error bars denote 95% confidence intervals

Figure 2a left panel presents the mean RTs for the baseline and learning groups. A mixed ANOVA on mean RTs with the within-subject factor distractor condition (distractor-present vs. -absent) and the between-subjects factor of group type (baseline vs. learning) showed an effect for distractor condition, *F*(1, 46) = 81.74, *p* < .001, *η*_*p*_^*2*^ = 0.64, but not for group type, *F*(1, 46) = 0.35, *p* = .556, *η*_*p*_^*2*^ = 0.08. Importantly, however, there was a significant interaction, *F*(1, 46) = 8.75, *p* = .005, *η*_*p*_^*2*^ = 0.16, showing that attentional capture was larger in the baseline group than in the learning group. Specifically, the attentional capture effect (AC effect; mean RTs in the distractor-absent condition minus that in the distractor-present condition) was larger in the baseline group (42 ms) than that in the learning group (21 ms). There were no effects on accuracy.

We divided the dataset into ten blocks. A mixed ANOVA on AC effect with the within-subject factor of block and the between-subjects factor of group type (baseline vs. learning) showed an effect for block, *F*(9, 414) = 3.65, *p* < .001, *η*_*p*_^*2*^ = 0.07, and group type, *F*(1, 46) = 9.49, *p* = .003, *η*_*p*_^*2*^ = 0.17, but no interaction was observed, *F*(9, 414) = 0.88, *p* = .542, *η*_*p*_^*2*^ = 0.02. It suggests that participants quickly learned the regularity and this started affecting the amount of capture already in block one (120 trials; *p* = .033, Cohen’s *d* = 0.64) and remained stable over the course of the experiment. In the present study, for each eight trials, the to-be-learned sequence was repeated. To determine how quickly participants had learned the regularity, we conducted an analysis based on each squence repetition (i.e., every eight trials) within block 1. The results showed that after presenting the sequence twice (16 trials), during the third sequence the capture effect was reliably smaller for the learning group than for the baseline, *t*(46) = 1.82, *p* = .041, Cohen’s *d* = 0.61. This indicates that learning is very fast (see also Wang & Theeuwes, 2020, for a similar finding).

When comparing distractor-present and -absent conditions across blocks of 120 trials, we found that in the learning group the attentional capture effect was no longer reliable in blocks 3, 6, 7, 8, and 9, all *p*s > .056 (see Fig. 2b).

After the experiment, participants had to indicate whether they noticed any regularity in the display, and if so, what this regularity was. None of the participants in the learning group reported that they detected any regularity regarding the learning sequence, suggesting that none of the participants had any explicit knowledge about the regularities introduced.

## Experiment 2

In Experiment 1 the color of the distractor was fixed across trials, which implies that the suppression may not have been spatial-based moving around in the display in a flexible way but instead feature-based, in which, regardless of its location, a particular color is proactively suppressed (Gaspelin et al., 2015). To test whether it is truly spatial-based instead of one distractor color we used two distractor colors that were randomly swapped between trials. In this way it was impossible to apply suppression that was feature (color) based.

### Method

Another group of 48 college students (14 men and 34 women: with a mean age of 19.8 ± 1.8 years old) participated in the present experiment. The procedure and the design were the same as those in Experiment 1, except that the distractor color was not fixed, but was swapped randomly across trials between red and green with equal probability.

### Results

Trials on which the RTs were faster than 200 ms or slower than 1,600 ms and trials on which no response was given (1.4% and 2.1% for baseline and learning groups, respectively) were excluded from analyses.

Figure 2a left panel presents the mean RTs for the baseline and learning groups. A mixed ANOVA on mean RTs with the within-subject factor of distractor condition (distractor-present vs. -absent) and the between-subjects factor of group type (baseline vs. learning) showed an effect for distractor condition, *F*(1, 46) = 193.08, *p* < .001, *η*_*p*_^*2*^ = 0.81, but not for group type, *F*(1, 46) = 3.5, *p* = .068, *η*_*p*_^*2*^ = 0.07. However, the critical interaction was highly reliable, *F*(1, 46) = 10.72, *p* = .002, *η*_*p*_^*2*^ = 0.19, as there was more attentional capture in the baseline group (48 ms) than in the learning group (29 ms). Moreover, when removing color-repeat trials, the interaction was observed as well, *p* = .028, *η*_*p*_^*2*^ = 0.1, with a larger attentional capture effect in the baseline trials (48 ms) than in the learning trials (35 ms). There were no effects on accuracy.

When dividing the dataset into ten blocks, a mixed ANOVA on AC effect with the within-subject factor of block and the between-subjects factor of group type (baseline vs. learning) showed an effect for block, *F*(9, 414) = 3.73, *p* < .001, *η*_*p*_^*2*^ = 0.08, and group type, *F*(1, 46) = 10.99, *p* = .002, *η*_*p*_^*2*^ = 0.19, but no interaction was observed, *F*(9, 414) = 0.72, *p* = .694, *η*_*p*_^*2*^ = 0.02. This suggests that, even though there were clear practice effects, the learning effect was stable across blocks (see Fig. 2b right panel). Similar to Experiment 1, after presenting the sequence twice (16 trials), there was a learning effect as there was reduced capture in the learning compared to the baseline group, *t*(46) = 2.26, *p* = .017, Cohen’s *d* = 0.72. For the learning group, the attentional capture effect was no longer reliable in block 5, *p* = .076.

After the experiment, participants were asked about whether they had noticed any regularity regarding the placement of the elements in the display. None of the participants in the learning group detected any regularities regarding the learning sequence, suggesting participants had no explicit knowledge regarding the regularities.

In the learning condition, the distractor always appeared adjacent to the distractor location on the previous trial. Previous studies have shown that repetition priming effects may not only be restricted to repeating the exact location of an item, but can also prime the item that is presented at the adjacent location (Geyer et al., 2010; Maljkovic & Nakayama, 1996). If, in the current paradigm, such an adjacent location-priming effect would be viable, then our explanation of statistical learning of a distractor sequence would be less surprising as “adjacent distractor” priming would be able to account for the effects observed. To exclude this possibility, we determined whether there was adjacent priming in the baseline condition. To gain statistical power we collapsed the baseline condition across the two experiments and analyzed trials in which the distractor on the current trial happened to be on the adjacent position on the previous trial. The analyses showed that there was no reliable across-adjacent-locations priming effect (8 ms, *p* = .701), suggesting that this across-adjacent distractor priming cannot explain our findings in the learning condition.

## General discussion

The present study shows a remarkable flexibility in attentional suppression. Even though participants were completely unaware, through statistical learning they extracted the spatial regularities regarding the distractor across trials. We show that the attentional system anticipates what will happen next, as the suppression is applied proactively to the anticipated location. Our findings show that this effect cannot be explained by across-adjacent location priming. Numerous previous studies have shown that we are able to avoid distraction by salient objects and events (e.g., Chang & Egeth, 2019, 2020; Feldmann-Wüstefeld et al., 2015; Failing et al., 2019; Ferrante et al., 2018; Gaspelin et al., 2015, 2017; Gaspelin & Luck, 2018a, b, 2019; Stilwell et al., 2019; van Moorselaar & Slagter, 2019; Vatterott & Vecera, 2012; Wang & Theeuwes, 2018a, 2018b; Won et al., 2019); here we show that this suppression is remarkably flexible. We argue that this suppression is spatial-based. Even though the results of Experiment 1 could be explained by assuming that one specific color feature was suppressed (e.g., the color red), this explanation does not hold for the findings of Experiment 2, in which the colors switched randomly from trial to trial.

As a mechanism, we assume that on any given trial, the suppression of a particular location generates a prediction regarding the location of the distractor on the next trial. Because of this prediction the anticipated location will be suppressed on the upcoming trial, reducing the effect of the distractor when it is presented there. We assume that the learning that we observed here affects the weights within the spatial priority map such that after suppressing a location on a given trial, the weight of the anticipated next location is down-regulated. This in turn results in less attentional capture of the salient distractor when it is presented at this proactively anticipated suppressed location. We assume that within the spatial priority map, the weights are combined into a single topographic representation of the environment (Fecteau & Munoz, 2006; Itti & Koch, 2001), which determines selection priority.

The current study shows that participants are able to learn to expect the location of the upcoming distractor, and, in turn, suppress this location such that attentional capture is reduced. Critically, in the current study the distractor followed a consistent clockwise or anticlockwise pattern in which the anticipated location was always adjacent to the current location. Whether participants are able to learn more complex patterns of anticipated distraction and/or anticipated enhancement (expecting a target at a particular location) is at this point unclear. Also, even if learning of more complex patterns is possible, it remains unclear whether the priority map can be tuned so flexibly across trials. Note, however, that recently it was shown that the priority map can even be tuned to particular moments in time within a trial. Xu et al. (in press) showed that participants learned to suppress one location after a short time interval (500 ms after fixation) and another location after a long time interval (1,500 ms after fixation). This study shows that participants can learn to suppress particular locations at particular moments in time, suggesting that the spatial priority map of attentional selection is highly flexible and can be dynamically adjusted during the trial.

It is unlikely that the flexible suppression that we observed here is due to an active (top-down) mechanism through which the anticipated location is suppressed. Even though some have suggested that top-down selection can be involuntary and unconscious (Gaspelin & Luck, 2018c), others have argued that a more precise definition is needed to make a distinction between active, voluntary selection and selection based on implicitly learned statistical regularities (Theeuwes, 2018a, 2018b). If one adheres to the position that one can only speak of top-down attention when observers are actively engaged, then the current findings cannot be explained in terms of top-down attention. For example, in a previous study, observers were asked to actively, in a top-down way, suppress the location of the upcoming distractor (Wang & Theeuwes, 2018b). The results indicated that observers were unable to suppress a location when they were asked to do so. Also, in the current study, none of the observers were aware of the regularities present in the display. If one is not aware of which location will contain the distractor on the next trial, it is hard to argue that this is an active top-down mechanism (see also Luck et al., 2021).

In the current study we measured awareness by asking, after the experiment was completed, whether participants were aware of any of the regularities, and if so, what these regularities were. This method of measuring awareness is the most direct method to find out whether a person is aware of a particular knowledge. This can be combined with additional subjective measures, such as confidence levels, in which participants indicate how confident they are about their responses (Timmermans & Cleeremans, 2015). It is obvious that this way of determining awareness has its limitations, as it is possible there is some level of awareness that cannot be verbalized (e.g., Dienes & Fahey, 1995). Indeed, Timmermans and Cleeremans (2015) provided an overview of various objective measures of awareness. Objective methods typically involve asking participants to choose between different alternatives (i.e., as in a two-alternative forced-choice task) instead of just asking about their thoughts. Even though in the current study the measurement of awareness was somewhat crude, it is unlikely that participants were aware of the regularity. In a previous study, in which we presented the distractor singleton consistently in the same location, less than half of the participants noticed the regularity even though it appeared at this high-probability location 13 times more often than in other locations (Wang & Theeuwes, 2018a). Given that in the current study the location of the distractor moves around constantly, it is highly unlikely that participants became aware of this.

Recently it has been argued that salient distractors may start interfering less because the attentional capture “respond” to a stimulus habituates (Turatto et al., 2019; Turatto & Pascucci, 2016). Indeed, because the same salient, irrelevant stimulus is repeatedly presented at one specific location, there may be a habituation of the attentional capture (Turatto & Pascucci, 2016). Even though this explanation may be feasible for previous findings in which the same singleton distractor was presented frequently in one location (e.g., Wang & Theeuwes, 2018a), a habituation explanation for the current findings is less likely because the salient stimulus moved around. Indeed, especially in our Experiment 2, there can be no habituation as the salient distractor moved around and had a color that changed randomly from trial to trial.

In our Experiment 1, in which the color of the distractor remained the same across trials, suppression could have been completely feature-based (Vatterott et al., 2018; Won et al., 2019). Yet, if suppression was completely feature-based, one would not have expected a difference between the learned and baseline groups as the feature that needs to be suppressed was basically the same in these different conditions. Therefore, the results of Experiment 1 can only be explained by a combination of spatial and feature-based suppression (e.g., Failing et al., 2019). However, Experiment 2 ruled out the contribution of feature-based suppression as the to-be-suppressed feature (the color of the distractor) varied randomly across trials. Note that we did not test the viability of feature-based suppression in the current study because it is highly unlikely that feature-based suppression played a role. Indeed, in a previous study it was shown that there was no contribution of feature-based suppression above and beyond spatial suppression (Wang & Theeuwes, 2018c). In Wang and Theeuwes (2018c, Experiments 3 and 4), the high-probability distractor location remained the same and the probability of the color of the distractor was varied. For example, for one group of participants, the distractor was red in 80% of trials and green in 20% of trials. The results showed no difference in the amount of suppression of the high-probability distractor location: regardless of whether it was a high- or low-probability color, the suppression was equally strong. Wang and Theeuwes (2018c) concluded that suppression induced by learned statistical regularities regarding the location of the distractor are not feature-specific. Given these previous findings, it is unlikely that feature-based suppression would play a role in the highly flexible suppression mechanism that we observed here.

Consistent with the idea that the distractor suppression is due to statistical learning, in the current study learning was extremely fast: as early as in block one, there was a learning effect, which did not change much over the course of the experiment. This rapid learning is consistent with previous studies that investigated statistical learning regarding the target probabilities (Ferrante et al., 2018; Jiang et al., 2013). We speculate that the learning is supported by the medial temporal lobe (MTL), in particular the hippocampus, as this brain region has been shown to allow a very fast extraction of regularities from the environment (Chun & Phelps, 1999; Turk-Browne et al., 2008; Turk-Browne et al., 2010).

The current study investigated whether participants can learn to anticipate the location of the salient distractor when the location of the distractor moves across the display. The current study shows participants have implicitly learned to anticipate what will happen next, and in anticipation of the presentation of the salient distract, suppression is proactively applied. The current findings are consistent with the notion that the spatial priority map is highly flexible and operates at a subconscious level preparing the attentional system for what will happen next. Future research should determine the boundary conditions investigating whether it is possible to learn more complex patterns of anticipated across-trial patterns of anticipated target and/or distractor locations.
